# Supplementary material for: HIVprotI: an integrated web based platform for prediction and design of HIV proteins inhibitors
Source: J Cheminform. 2018 Mar 9;10:12. doi: 10.1186/s13321-018-0266-y (PMC5845081; doi:10.1186/s13321-018-0266-y)
Supplement: Supplementary file 2 — Additional file 2. Source code of HIVProtI web server. [file 13321_2018_266_MOESM2_ESM.zip › HIVprotI_Source-code/hivprotI/weka-3-6-6/doc/weka/gui/visualize/PrintableComponent.html]

PrintableComponent


---


|  |  |  |
| --- | --- | --- |
| **Overview** \| **Package** \| **Class** \| **Tree** \| **Deprecated** \| **Index** \| **Help** \| **Weka's home** | |  |
| **PREV CLASS**   **NEXT CLASS** | **FRAMES**    **NO FRAMES**     **All Classes** |  |
| SUMMARY: NESTED \| FIELD \| CONSTR \| METHOD | DETAIL: FIELD \| CONSTR \| METHOD |  |


---


## weka.gui.visualize Class PrintableComponent

```
java.lang.Object
  weka.gui.visualize.PrintableComponent
```

**All Implemented Interfaces:**: PrintableHandler

---

``` public class PrintableComponent extends java.lang.Object implements PrintableHandler ```

This class extends the component which is handed over in the constructor
by a print dialog.
The Print dialog is accessible via Alt+Shift+LeftMouseClick.

The individual JComponentWriter-descendants can be accessed by the
`getWriter(String)` method, if the parameters need to be changed.

**Version:**
:   $Revision: 7059 $

**Author:**
:   FracPete (fracpete at waikato dot ac dot nz)

**See Also:**: `getWriters()`, `getWriter(String)`

---

**Constructor Summary**

`PrintableComponent(javax.swing.JComponent component)`
  
          initializes the panel.


| **Method Summary** | |
| --- | --- |
| `javax.swing.JComponent` | `getComponent()`             returns the GUI component this print dialog is part of. |
| `java.lang.String` | `getSaveDialogTitle()`             returns the title for the save dialog. |
| `static java.lang.String` | `getToolTipText(PrintableComponent component)`             Returns a tooltip only if the user wants it. |
| `JComponentWriter` | `getWriter(java.lang.String name)`             returns the JComponentWriter associated with the given name, is `null` if not found. |
| `java.util.Hashtable` | `getWriters()`             returns a Hashtable with the current available JComponentWriters in the save dialog. |
| `double` | `getXScale()`             returns the scale factor for the x-axis. |
| `double` | `getYScale()`             returns the scale factor for the y-axis. |
| `void` | `saveComponent()`             displays a save dialog for saving the panel to a file. |
| `void` | `setSaveDialogTitle(java.lang.String title)`             sets the title for the save dialog. |
| `void` | `setScale(double x, double y)`             sets the scale factor. |

**Methods inherited from class java.lang.Object**

`equals, getClass, hashCode, notify, notifyAll, toString, wait, wait, wait`

**Constructor Detail**

### PrintableComponent

```
public PrintableComponent(javax.swing.JComponent component)
```

:   initializes the panel.

    **Parameters:**: `component` - the component to enhance with printing functionality


**Method Detail**

### getComponent

```
public javax.swing.JComponent getComponent()
```

:   returns the GUI component this print dialog is part of.

    :   **Returns:**: the GUI component

---


### getToolTipText

```
public static java.lang.String getToolTipText(PrintableComponent component)
```

:   Returns a tooltip only if the user wants it. If retrieved for the first,
    a dialog pops up and asks the user whether the tooltip should always
    appear or not. The weka/gui/visualize/Visualize.props is then written
    in the user's home directory.

    :   **Parameters:**: `component` - the PrintableComponent to ask for **Returns:**: null if the user doesn't want the tooltip, otherwise the text

---


### getWriters

```
public java.util.Hashtable getWriters()
```

:   returns a Hashtable with the current available JComponentWriters in the
    save dialog. the key of the Hashtable is the description of the writer.

    :   **Specified by:**: `getWriters` in interface `PrintableHandler`
    :   **Returns:**: all currently available JComponentWriters **See Also:**: `JComponentWriter.getDescription()`

---


### getWriter

```
public JComponentWriter getWriter(java.lang.String name)
```

:   returns the JComponentWriter associated with the given name, is
    `null` if not found.

    :   **Specified by:**: `getWriter` in interface `PrintableHandler`
    :   **Parameters:**: `name` - the name of the writer **Returns:**: the writer associated with the given name **See Also:**: `JComponentWriter.getDescription()`

---


### setSaveDialogTitle

```
public void setSaveDialogTitle(java.lang.String title)
```

:   sets the title for the save dialog.

    :   **Specified by:**: `setSaveDialogTitle` in interface `PrintableHandler`
    :   **Parameters:**: `title` - the title of the save dialog

---


### getSaveDialogTitle

```
public java.lang.String getSaveDialogTitle()
```

:   returns the title for the save dialog.

    :   **Specified by:**: `getSaveDialogTitle` in interface `PrintableHandler`
    :   **Returns:**: the title of the save dialog

---


### setScale

```
public void setScale(double x,
                     double y)
```

:   sets the scale factor.

    :   **Specified by:**: `setScale` in interface `PrintableHandler`
    :   **Parameters:**: `x` - the scale factor for the x-axis: `y` - the scale factor for the y-axis

---


### getXScale

```
public double getXScale()
```

:   returns the scale factor for the x-axis.

    :   **Specified by:**: `getXScale` in interface `PrintableHandler`
    :   **Returns:**: the scale factor

---


### getYScale

```
public double getYScale()
```

:   returns the scale factor for the y-axis.

    :   **Specified by:**: `getYScale` in interface `PrintableHandler`
    :   **Returns:**: the scale factor

---


### saveComponent

```
public void saveComponent()
```

:   displays a save dialog for saving the panel to a file.
    Fixes a bug with the Swing JFileChooser: if you entered a new
    filename in the save dialog and press Enter the `getSelectedFile`
    method returns `null` instead of the filename.  
    To solve this annoying behavior we call the save dialog once again s.t. the
    filename is set. Might look a little bit strange to the user, but no
    NullPointerException! ;-)

    :   **Specified by:**: `saveComponent` in interface `PrintableHandler`


---


|  |  |  |
| --- | --- | --- |
| **Overview** \| **Package** \| **Class** \| **Tree** \| **Deprecated** \| **Index** \| **Help** \| **Weka's home** | |  |
| **PREV CLASS**   **NEXT CLASS** | **FRAMES**    **NO FRAMES**     **All Classes** |  |
| SUMMARY: NESTED \| FIELD \| CONSTR \| METHOD | DETAIL: FIELD \| CONSTR \| METHOD |  |


---
